# Molecular Epidemiology of Shiga Toxin-Producing Escherichia coli (STEC) on New Zealand Dairy Farms: Application of a Culture-Independent Assay and Whole-Genome Sequencing

**DOI:** 10.1128/AEM.00481-18

**Published:** 2018-07-02

**Authors:** A. Springer Browne, Anne C. Midwinter, Helen Withers, Adrian L. Cookson, Patrick J. Biggs, Jonathan C. Marshall, Jackie Benschop, Steve Hathaway, Neville A. Haack, Rukhshana N. Akhter, Nigel P. French

**Affiliations:** a *^m^*EpiLab, Massey University, Palmerston North, New Zealand; b Ministry of Primary Industries, Wellington, New Zealand; c AgResearch Limited, Palmerston North, New Zealand; d New Zealand Food Safety Science & Research Centre, Palmerston North, New Zealand; INRS—Institut Armand-Frappier

**Keywords:** New Zealand, STEC, Shiga-toxigenic Escherichia coli, cattle, cross-sectional studies

## Abstract

New Zealand has a relatively high incidence of human cases of Shiga toxin-producing Escherichia coli (STEC), with 8.9 STEC cases per 100,000 people reported in 2016. Previous research showed living near cattle and contact with cattle feces as significant risk factors for STEC infections in humans in New Zealand, but infection was not linked to food-associated factors. During the 2014 spring calving season, a random, stratified, cross-sectional study of dairy farms (*n* = 102) in six regions across New Zealand assessed the prevalence of the “Top 7” STEC bacteria (serogroups O157, O26, O45, O103, O111, O121, and O145) in young calves (*n* = 1,508), using a culture-independent diagnostic test (PCR/MALDI-TOF). Twenty percent (306/1,508) of calves on 75% (76/102) of dairy farms were positive for at least one of the “Top 7” STEC bacteria. STEC carriage by calves was associated with environmental factors, increased calf age, region, and increased number of calves in a shared calf pen. The intraclass correlation coefficient (ρ) indicated strong clustering of “Top 7” STEC-positive calves for O157, O26, and O45 serogroups within the same pens and farms, indicating that if one calf was positive, others in the same environment were likely to be positive as well. This finding was further evaluated with whole-genome sequencing, which indicated that a single E. coli O26 clonal strain could be found in calves in the same pen or farm, but different strains existed on different farms. This study provides evidence that would be useful for designing on-farm interventions to reduce direct and indirect human exposure to STEC bacteria.

**IMPORTANCE** Cattle are asymptomatic carriers of Shiga toxin-producing E. coli (STEC) bacteria that can cause bloody diarrhea and kidney failure in humans if ingested. New Zealand has relatively high numbers of STEC cases, and contact with cattle feces and living near cattle are risk factors for human infection. This study assessed the national prevalence of STEC in young dairy cattle by randomly selecting 102 farms throughout New Zealand. The study used a molecular laboratory method that has relatively high sensitivity and specificity compared to traditional methods. “Top 7” STEC was found in 20% of calves on 75% of the farms studied, indicating widespread prevalence across the country. By examining the risk factors associated with calf carriage, potential interventions that could decrease the prevalence of “Top 7” STEC bacteria at the farm level were identified, which could benefit both public health and food safety.

## INTRODUCTION

Worldwide, Shiga toxin-producing Escherichia coli (STEC) bacteria are a growing public health concern. Large-scale outbreaks in Europe (1) and the United States (see https://www.cdc.gov/ecoli/2015/o26-11-15) have continued to occur. Furthermore, human STEC cases in Argentina have a high rate of serious clinical complications (2). Although STEC cases may have a lower prevalence than other notifiable zoonotic diseases (3), the pathogen's propensity to affect very young children, leading to potential long-term kidney and brain damage (4), is a concerning public health issue. STEC bacteria are primarily transmitted via the fecal-oral route. Ruminant animals, particularly cattle, have been identified as the most important reservoir (5).

New Zealand has a relatively high incidence of notified STEC infection in humans, with 8.9 STEC cases per 100,000 people reported in 2016 (6), compared to 2.85 per 100,000 in the United States in 2016 (7), and 12.92 in Ireland, 5.08 in the Netherlands, and 2.05 per 100,000 in the United Kingdom in 2015 (https://ecdc.europa.eu/en/escherichia-coli-ecoli/surveillance/atlas). Since it became a notifiable disease in New Zealand, there has been a general increase of STEC cases annually, with both STEC O157:H7 and non-O157 STEC causing human disease (8). A New Zealand case-control study identified contact with animal manure and the presence of cattle in the local area, along with contact with recreational waters, as significant risk factors for human STEC infection (9). Interestingly, the same study did not identify food as a statistically significant exposure pathway in New Zealand (9). Previous research findings overseas have highlighted beef food products and raw produce as the main sources of human infection (5, 10, 11), but findings in the United Kingdom also identified an important contribution from environmental sources (12). Determining the carriage of STEC in ruminant hosts through targeted national studies will help our understanding of the epidemiology of this important pathogen.

Since the 1993 outbreak of STEC O157:H7 in the United States (13), monitoring and regulatory requirements regarding this pathogen have increased. After finding STEC O157:H7 in raw ground beef and the occurrence of outbreaks associated with consumption of undercooked beef patties, the United States declared STEC O157:H7 an adulterant of beef in 1994, followed in 2011 by the declaration of six additional serogroups (O26, O45, O103, O111, O121, and O145) as adulterants (14). These six additional serogroups and STEC O157 are known as the “Top 7” STEC. In 2015 to 2016, 50% of New Zealand beef exports were sent to the United States (15). Given the importance of agricultural exports for the New Zealand economy, STEC is an economic as well as a public health concern.

Previous research in New Zealand identified a higher prevalence of STEC O157 and STEC O26 in young calves than in adult cattle (16), and this finding has been supported by “Top 7” STEC research in other countries (17–20). New Zealand dairy farms follow a seasonal calving strategy in which surplus dairy calves, known as bobby calves, may be slaughtered at a very young age (4 to 10 days old). The higher prevalence of STEC among very young calves means that preventing inadvertent contamination of veal during dressing of carcasses at primary processing is an important risk management goal. Similarly, reducing children's contact with calves may lessen the risk to human health.

This study examined the prevalence of young calves shedding “Top 7” STEC bacteria (serogroups O157, O26, O45, O103, O111, O121, and O145) on dairy farms in New Zealand. We estimated the spatial distribution of STEC-positive farms and determined the clonal relationships of STEC bacteria in calves by pen and farm and risk factors for STEC carriage by calves that could potentially be targeted for control. By understanding and reducing STEC from its source, we hope to decrease the risk of both veal meat contamination and human exposure to STEC on farms.

## RESULTS

### Prevalence of “Top 7” serogroups determined by latent class analysis of real-time PCR and NeoSEEK.

**FIG 1:**
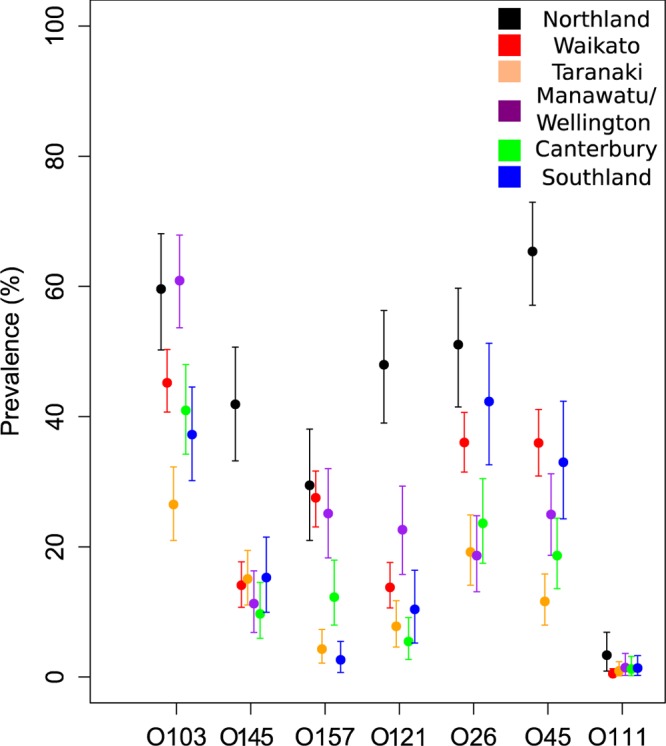
“Top 7” serogroup prevalence (with 95% CI), including both STEC and non-STEC isolates, detected in calves (*n* = 1,508) by region, using latent class analysis of NeoSEEK and real-time PCR results.

**FIG 2:**
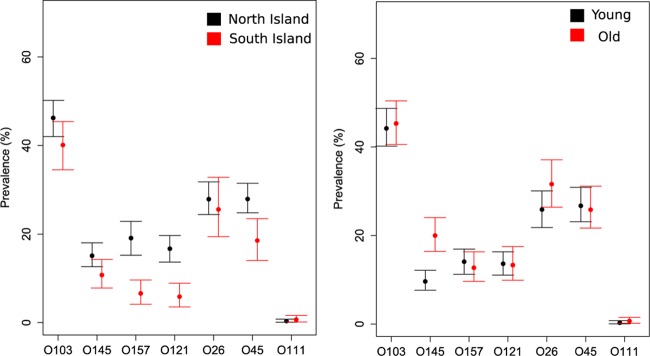
“Top 7” serogroup prevalence (with 95% CI), including both STEC and non-STEC isolates, detected in calves (*n* = 1,508) by island and age (young, 2 to 9 days; old, 10 to 21 days), using latent class analysis of NeoSEEK and real-time PCR results.

Our in-house real-time PCR assay was only able to detect the presence of the O serogroup in a sample, while the NeoSEEK assay claims to be able to discriminate between *stx*-positive and *stx*-negative E. coli of a “Top 7” STEC serogroup (e.g., STEC O26 versus nontoxigenic O26). By using latent class modeling techniques, the prevalence of these serogroups was determined, using both assays to give a more robust estimation of serogroup prevalence (Fig. 1 and 2). This modeling technique required the calf population to be divided into groups for comparison; we therefore stratified by region, by presence on North and/or South Island, and by age (young, 2 to 9 days; old, 10 to 21 days).

There were notable differences in estimated serogroup prevalence between groups. Northland, Manawatu-Wellington, and Waikato had a high prevalence of several “Top 7” STEC serogroups compared to that of other regions, particularly of serogroups O26 and O45 (Fig. 1). Prevalence between older and younger calves was similar, but older calves had a higher prevalence of O145 and O26 serogroups (Fig. 2). Finally, the North Island had a higher prevalence of most serogroups, with the exception of O26, for which the two islands had similar prevalences (Fig. 2).

### “Top 7” STEC detection via culture-independent methods.

**FIG 3:**
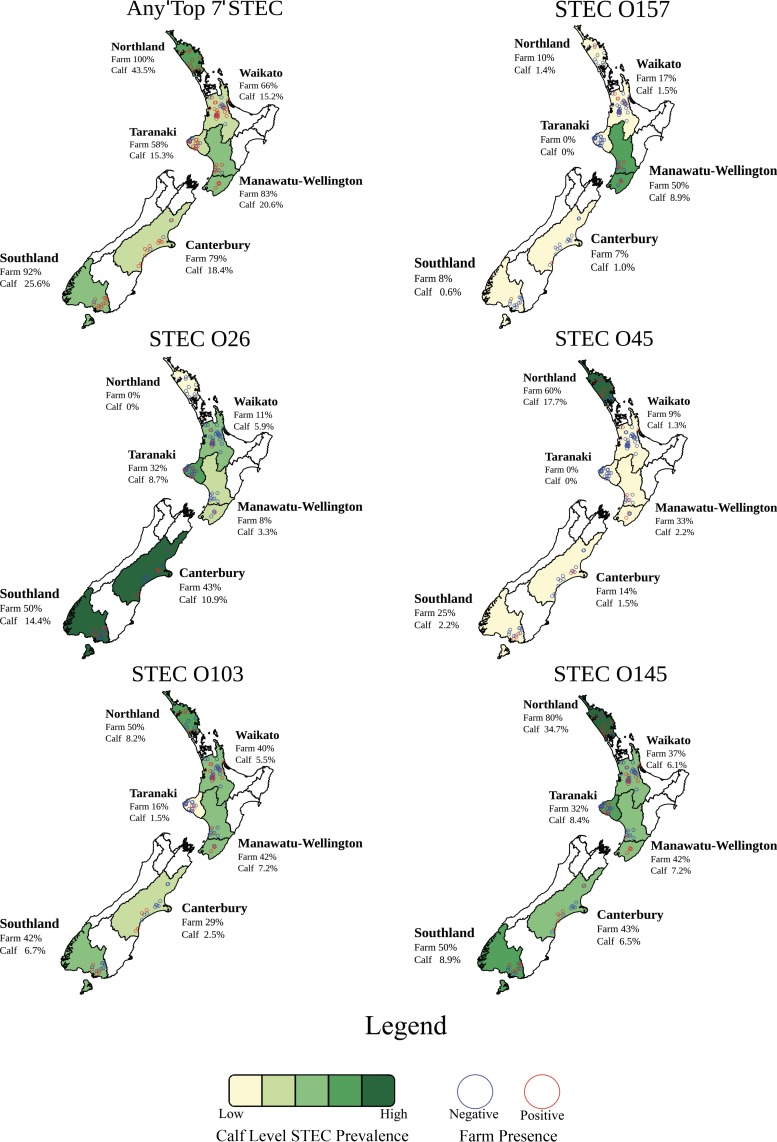
Calf-level (*n* = 1,508) and farm-level (*n* = 102) prevalence of the “Top 7” STEC on New Zealand dairy farms by region (*n* = 6).

NeoSEEK detected 20.3% (95% confidence interval [CI], 16.1 to 24.5) of the calves on 75% (76/102) of the dairy farms as positive for at least one of the “Top 7” STEC (Table 1). NeoSEEK identifies both the presence of a “Top 7” serogroup, as well as the presence of *eae* and *stx* genes, within the same serogroup, using PCR/matrix-assisted laser desorption ionization–time of flight mass spectrometry (MALDI-TOF). All “Top 7” STEC serogroups, except for STEC O121, were detected in samples taken from the New Zealand dairy farms tested. The highest estimated STEC serogroup prevalences at the farm and calf level were STEC O145 and STEC O26, while STEC O111 was only detected in recto-anal mucosal swabs (RAMS) from three calves located on one farm in the Northland region. Prevalence maps illustrate the regional variability of prevalence of “Top 7” STEC in New Zealand (Fig. 3). “Top 7” STEC prevalence varied between serogroups, with STEC O26 more commonly detected in the South Island (Canterbury and Southland) and a much higher prevalence of STEC O45 detected in Northland compared to other regions.

**TABLE 1:** Farm-level (*n* = 102) and calf-level (*n* = 1,508) prevalence of the “Top 7” STEC on New Zealand dairy farms

Prevalence	Value for serogroup(s)							
	STEC O103	STEC O121	STEC O111	STEC O145	STEC O157	STEC O26	STEC O45	Any “Top 7” STEC^*a*^
Calves								
No. positive	75	0	3	148	29	109	44	306
% positive (95% CI)	5.0 (2.7–7.2)	0	0.2 (0.0–0.6)	9.8 (6.7–12.9)	1.9 (0.5–3.3)	7.2 (4.5–9.9)	2.9 (1.2–4.7)	20.3 (16.1–24.5)
Farms								
No. positive	36	0	1	44	15	23	18	76
% positive	35	0	1	43	15	23	18	75

a The detection of at least one of the “Top 7“ STEC bacteria in an individual calf. A total of 408 instances of “Top 7“ STEC were detected, but some calves shed multiple STEC serogroups, as follows: 1 serogroup (*n* = 217), 2 serogroups (*n* = 76), and 3 serogroups (*n* = 13) (see Table S1 in the supplemental material).

The virulence genes *stx* and *eae* were common in calf samples; NeoSEEK detected *stx* in 70.5% of the calf samples and *eae* in 57.6% of calf samples. Both *eae* and *stx* genes were detected in 45.4% of calf samples; however, it is important to note that this did not necessarily indicate that a “Top 7” STEC was present. The *stx* gene was detected in at least one calf sample from all farms in the study, while *eae* was detected in at least one calf sample from 101 of the 102 farms.

Estimation of the intraclass correlation coefficient (ρ) was calculated based on the presence or absence of a “Top 7” STEC-positive calf in a particular pen or farm. The intraclass correlation coefficient (ρ) revealed strong clustering of “Top 7” STEC-positive calves within pens and some strong clustering of calves on farms, most notably with the STEC O26, STEC O157, and STEC O45 serogroups (Table 2).

**TABLE 2:** Intraclass correlation coefficient (ρ) values of “Top 7” STEC using farm (*n* = 102) and calf pen (*n* = 267) as a random factor

Factor evaluated	ρ value					
	STEC O103	STEC O145	STEC O157	STEC O26	STEC O45	Any “Top 7” STEC
Farm	0.13	0.29	0.61^*a*^	0.68^*a*^	0.62^*a*^	0.24
Calf pen	0.57	0.60^*a*^	0.71^*a*^	0.79^*a*^	0.77^*a*^	0.34

a Strong clustering observed.

Calf- and farm-level risk factors were evaluated for the three most prevalent STEC serogroups (STEC O26, STEC O103, and STEC O145) and the presence of any “Top 7” STEC. Due to the low calf-level prevalence of STEC O157 (*n* = 29 calves), STEC O45 (*n* = 44 calves), and STEC O111 (*n* = 3 calves), it was not possible to create a final model using the same statistical technique for these serogroups; therefore significant risk factors were not identified. Region, higher humidity measured inside the calf pen compared to that outside the calf housing area, older calf age, and increased number of calves in a pen were all identified as significant risk factors for the presence of any “Top 7” STEC (Table 3). Individual STEC serogroup analysis revealed increased number of calves in a pen (STEC O26; see Table S2 in the supplemental material), increased pen humidity and a high ammonia presence (determined subjectively) in a pen (STEC O103; see Table S3), and region, increased age, and increased pen humidity (STEC O145; see Table S4) as significant risk factors.

**TABLE 3:** Logistic mixed effects regression model^*j*^ of factors associated with prevalence of any “Top 7” STEC

Factor evaluated	OR^*a*^	95% CI	*P* value
Humidity^*b*^	1.09	1.02–1.16	0.006^*g*^
Region^*c*^			0.001^*i*^
Waikato	0.09	0.03–0.29	<0.001^*g*^
Taranaki	0.11	0.03–0.39	<0.001^*g*^
Manawatu-Wellington	0.23	0.06–0.87	0.030^*g*^
Canterbury	0.19	0.05–0.72	0.014^*g*^
Southland	0.30	0.08–1.13	0.076
No. of calves in calf pen^*d*^	1.04	1.01–1.07	0.003^*g*^
Temperature^*e*^	1.20	0.96–1.49	0.114^*h*^
Age^*f*^	0.43	0.27–0.68	<0.001^*g*^

a OR, odds ratio.

b Difference between inside pen versus outside the calf housing area (increase in 1% relative humidity).

c Compared to Northland.

d Increase of one calf.

e Difference between inside pen versus outside the calf housing area (increase of 1°C).

f Young calves (2 to 9 days of age) versus older calves (10 to 21 days of age).

g Significant variable (*P* < 0.05).

h Confounding factor for calf pen humidity, left in model.

i Likelihood ratio test of factor.

j Variance of random effects: calf pen within farm (variance, 1.09); farm (variance, 1.34).

### Bacterial isolation of E. coli serogroup O26 and O157.

A total of 31 STEC O157 isolates, 123 STEC O26 isolates, and 69 nontoxigenic O26 isolates were retrieved from 138 calf fecal enrichment broths. The results of bacterial isolation of O157 and O26 E. coli serogroups from calf fecal enrichment broths (samples) are shown in Table 4, where results are based on the successful recovery or failure of recovery of at least one isolate from a calf fecal enrichment broth.

**TABLE 4:** Bacterial isolation of STEC and non-STEC isolates of serogroup O157 and O26 E. coli from calf fecal enrichment broths

Serogroup	No. of samples detected as STEC by NeoSEEK	No. of samples from which an isolate was recovered/total no. of samples (%)^*a*^	No. of samples from which an STEC isolate was recovered/no. of samples from which an isolate was recovered (%)^*a*^	No. of samples from which an STEC isolate was recovered/total no. of samples (%)
O157	29	14/29 (48)	14/14 (100)	14/29 (48)
O26	109	70/109 (64)	49/70 (70)	49/109 (45)

a At least one isolate was recovered from the enrichment broth.

### Whole-genome sequencing (WGS) of serogroup O26 bacterial isolates.

**FIG 4:**
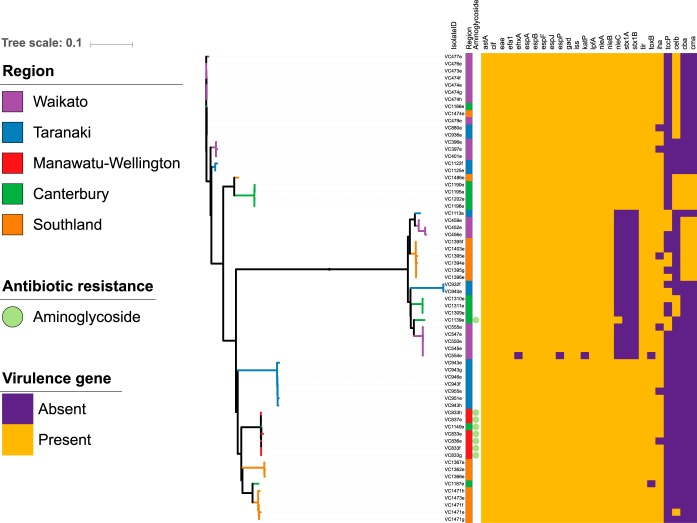
Maximum-likelihood core genome tree of serogroup O26 calf isolates (*n* = 66), annotated with region (*n* = 6), antibiotic resistance gene class (*n* = 1), and virulence genes (*n* = 26).

**FIG 5:**
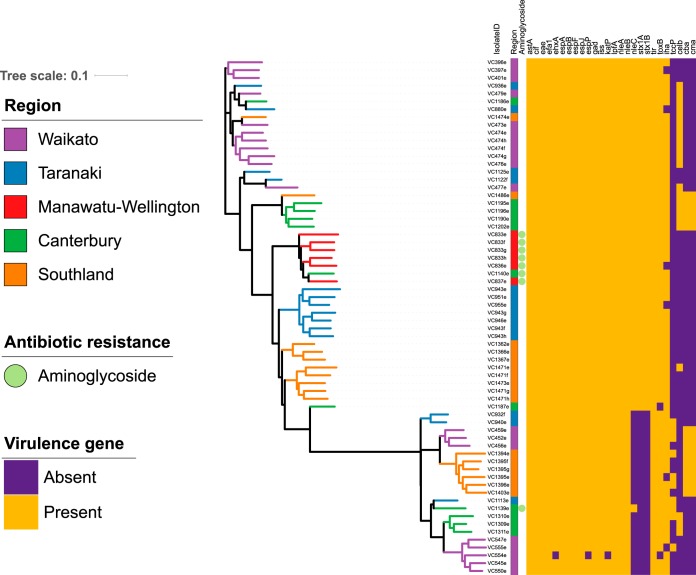
Maximum-likelihood accessory genome tree of serogroup O26 calf isolates (*n* = 66), annotated with region (*n* = 6), antibiotic resistance gene class (*n* = 1), and virulence genes (*n* = 26).

WGS data of serogroup O26 isolates (*n* = 66, 45/66 STEC O26) from 24 sheds on 18 farms in five regions of New Zealand were processed using the Nullarbor pipeline and the Center for Genomic Epidemiology output to evaluate the core genome, accessory genome, virulence genes, and antibiotic resistance genes (21, 22). The core genome (Fig. 4) and accessory genome (Fig. 5) were annotated with region, antimicrobial resistance gene class (*n* = 1), and virulence gene (*n* = 26) presence or absence. Clear clustering of STEC O26 isolates (*n* = 45) distinct from nontoxigenic isolates (*n* = 21) was visible in both Figure 4 and 5, but no obvious clustering by region was seen. The heatmap of virulence genes detected (*n* = 26) indicated that STEC O26 and nontoxigenic serogroup O26 E. coli had distinct virulence gene profiles (Fig. 4 and 5). Antimicrobial resistance gene detection was rare, with only aminoglycoside resistance class genes detected [*strA*, *strB*, *aph(3′)-IIa*-like] in eight isolates from the Manawatu-Wellington and Canterbury regions. All genomes sequenced from O26 bacterial isolates retrieved from calves in this study were identified as multilocus sequence type 21 (ST21), and serotype O26:H11.

Single nucleotide polymorphism analysis between serogroup O26 isolates indicated that the same clonal strain existed in calves in the same pen and the same farm, while strains between farms were different (see Fig. S1 in the supplemental material; total single nucleotide polymorphisms [SNPs], 11,167). For analysis of isolates from calves (*n* = 42) on the same farm (*n* = 14), as well as in the same pens (*n* = 20), 0 to 29 SNPs separated all isolates. A subset of calves (*n* = 5) had multiple isolates (*n* = 19 total, range 3 to 4 isolates from the same animal) sequenced from the same animal; only 0 to 17 SNPs separated isolates retrieved from the same animal sample. Two exceptions were noted in the analysis, where two calves had markedly different (by 214 SNPs and 223 SNPs) O26 strains compared to those in other calves in the same farm and pen, indicating that multiple serogroup O26 strains were present in the farm environment at the same time.

**FIG 6:**
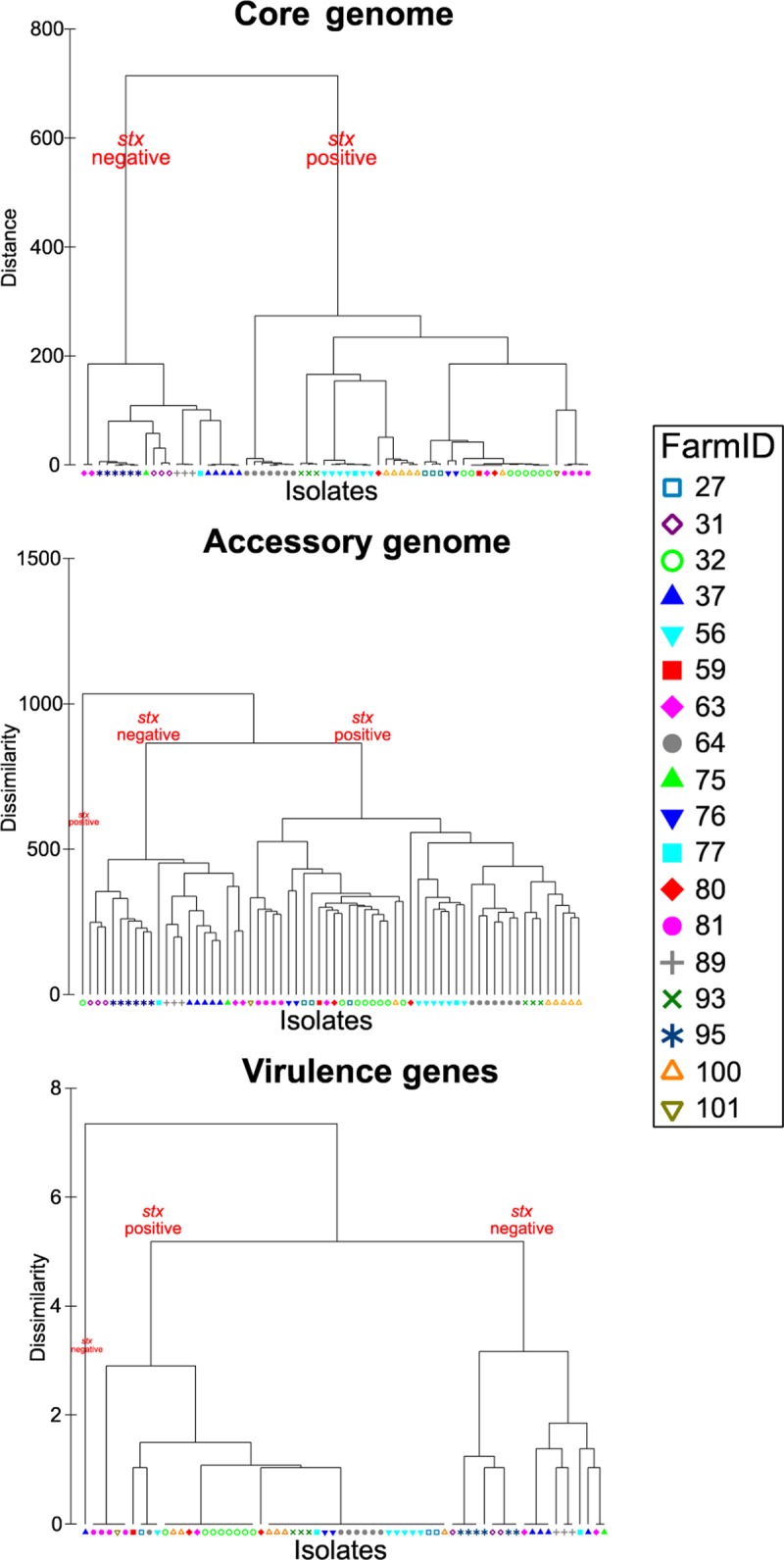
Hierarchical cluster trees of core, accessory, and virulence genes by farm (*n* = 18).

Permutational multivariate analysis of variance (PERMANOVA) analysis was used to compare region and farm with the variabilities of the core genome (SNP distance), accessory genome (presence or absence of accessory genes), and virulence genes (presence or absence of virulence genes) (Table 5). Farm was a significant predictor of variability (69.7 to 88.5%), indicating that the majority of the genetic variability at the core, accessory, and virulence gene levels could be associated with each calf's presence in a specific farm environment. The importance of farm was further evaluated in hierarchical cluster plots (Fig. 6), where a clear differentiation based on farm is visible, with the exception of farms which contain both *stx*-positive and *stx*-negative isolates. The hierarchical cluster analysis of core, accessory and virulence gene profiles also separated *stx*-positive and *stx*-negative isolates into different clonal groups, despite all being the same multilocus sequence type (ST21).

**TABLE 5:** PERMANOVA analysis of core genome (SNP distance matrix), accessory genome (presence or absence of accessory genes), and virulence genes by region (*n* = 5) and farm (*n* = 18)

Factor evaluated	Genomic data set	df	Pseudo-F	*P* value	Component of variation (%)^*a*^
Region	Core	4	1.9	0.0975	NS
	Accessory	4	2.69	0.0016	11.6
	Virulence	4	1.36	0.245	NS
Farm	Core	17	28.6	0.0001	88.5
	Accessory	17	9.3	0.0001	69.7
	Virulence	17	24.7	0.0001	86.8

a Residual variation: core (11.5%), accessory (30.2%), and virulence (13.2%). NS, not significant.

## DISCUSSION

This study utilized an established molecular method that distinguishes STEC and non-STEC variants, along with random stratified sampling, to estimate the national prevalence of the “Top 7” STEC in young calves on dairy farms throughout New Zealand. Statistical analyses evaluated risk factors for positive prevalence in calves, while WGS and further statistical analysis determined the similarity of “Top 7” STEC isolates between calves in a shared environment.

Systematic review and meta-analysis estimated an 8.7% prevalence of STEC (both *eae* and *stx* present in a single bacterium) in calves from 19 countries (23). A comprehensive national prevalence study of cattle and calves at 31 Australian processing plants using culture methods showed a 6.3% prevalence of STEC O157, with a 1.7% prevalence for the non-O157 “Top 7” STEC (17). This Australian study also found that veal calves had the highest potential STEC prevalence (12.7%), compared to that in young beef, young dairy, and adult cattle, with 51% of all samples testing positive for both *eae* and *stx* virulence markers via PCR methods (17). Our results indicated a higher “Top 7” STEC prevalence (20.3%) in young calves; our use of a culture-independent diagnostic test may have increased the sensitivity of detection of STEC.

Several results in our analysis suggested that STEC transmission occurs between calves or within the immediate calf pen environment: a high intraclass correlation coefficient (ρ) indicated strong clustering of “Top 7” STEC-positive calves in pens for STEC O26, STEC O157, and STEC O45; an increased risk of “Top 7” STEC prevalence with increasing numbers of calves in a single pen; and evidence of clonal strains of serogroup O26 E. coli observed in specific farms and pens. In a controlled transmission study, a calf infected with a low dose of STEC O157 began shedding the bacteria within 6 days, and STEC O157 subsequently colonized all other calves in the same pen within 4 to 11 days after the initial calf began shedding (24). A separate comparison study of calves housed in individual pens versus in an open group pen showed that a single calf inoculated with a control STEC strain in a group pen infected all other calves in that group over 10 days (25). Modeling studies have deduced an R_0_ (basic reproduction number) of 4.3 to 7.3 for STEC O157 in young calves from both natural and induced infection, suggesting that calves in shared environments infect numerous other individuals when shedding (26, 27). The observed clustering was most likely due to transmission of STEC in the immediate environment, but other factors at the pen and farm level may explain these findings.

Our WGS analysis indicated that *stx*-positive and *stx*-negative E. coli O26 bacteria form distinct clones with divergent core, accessory, and virulence gene profiles. Further epidemiological analysis also demonstrated that unique E. coli O26 clones disseminate among calves in a farm environment. PERMANOVA results indicated that farm, but not region, was a significant predictor of genetic variability (Table 5). The lack of similarity among strains in the same region, as well as the similarity between isolates on farms, suggests relatively low transmission between farms in the same region. Only a minority of farms sampled in this study had brought animals from outside the farm onto their property in the past two calving seasons: 7/102 farms had brought in calves, while 15/102 farms had brought in adult cows. It is likely that, once established, specific strains proliferate on farms, leading to transmission between animals on the same farm. This finding has been reflected in other studies, where STEC strains isolated from calves from the same pen showed low variability, indicating frequent within-pen transmission (28). Unique STEC O157 lineages also proliferated among cattle on US dairy farms with a high STEC O157 prevalence (29). SNP analysis indicated that STEC O157 populations on a farm were dominated by a single clonal type, but differences occurred between farms, and some clonal types were still present during resampling 11 months later (29). Pulsed-field gel electrophoresis (PFGE) analysis of O26:H11 isolates (*n* = 11) on three Australian farms also found unique strains at each farm (30).

Increased relative humidity inside the pen environment compared to that outside the calf housing area was associated with increased “Top 7” STEC prevalence. Higher humidity has been associated with increased risk of shedding STEC O157 bacteria (31), but it is unclear whether this is due to environmental factors that would benefit bacterial growth or to high humidity causing stress to the animal. The increase in STEC prevalence with calf age may be associated with the duration of STEC exposure within the pen. The longer the calf is present with other infected animals in an STEC-contaminated environment, the more the likelihood of STEC ingestion and colonization increases.

The calf pen environment is an important potential intervention point. Decreasing the number of young calves in pens is a practical intervention that may decrease STEC carriage. This may also have animal welfare benefits. Recent legislation in New Zealand (see http://www.legislation.govt.nz/regulation/public/2018/0050/latest/whole.html) has focused on young calf welfare, and mandatory management changes could lead to opportunities for interventions at the farm level. Individual outdoor calf hutches, although used in other countries, are not widely used in New Zealand and may not be a realistic intervention for dairy farmers from either a time management or an economic viewpoint.

Limitations of this study included the cross-sectional study design, which estimated STEC prevalence based on a single sampling event. It is well documented that calves may shed STEC intermittently, showing daily or even hourly variations (32, 33). By sampling many calves from multiple pens on each farm, we estimated the farm level prevalence, as well as the proportion of calves shedding “Top 7” STEC bacteria on a dairy farm at a single point in time.

The utilization of culture-independent diagnostic tests (CIDTs) to detect bacterial zoonoses is rapidly increasing, due to lower costs and increased speed of detection compared to traditional culture methods; CIDTs for detecting STEC are widely employed (34). However, our use of a culture-independent diagnostic test (NeoSEEK) for this epidemiological study may have led to false positives, due to a less than 100% specificity compared to that of culture methods. We evaluated the assay on New Zealand “Top 7” STEC isolates, and several other USDA studies in the United States have shown successful bacterial isolation of 84% (61/73) “Top 7” STEC isolates (20) and 55.7% (305/548) of non-STEC isolates (35) following “Top 7” STEC detection using the NeoSEEK assay. The New Zealand Ministry of Primary Industries has approved and utilized NeoSEEK as part of the regulatory testing and holding program for veal beef exports to the United States, and the use of the NeoSEEK assay in a research context was beneficial for this epidemiological research.

### Conclusion.

This cross-sectional study of young calves on New Zealand dairy farms identified the widespread presence of “Top 7” STEC bacteria. Future work using similar molecular confirmation methods, along with WGS, will permit the evaluation of the transmission dynamics of the “Top 7” STEC on New Zealand dairy farms by sampling calves, cows, and their immediate environment throughout the calving season. Data from this research will provide further information as to the importance of specific environmental sources of infection for calves, as well as the persistence and spread of STEC throughout the dairy farm environment.

Practical and economic factors are often key drivers influencing the uptake and adoption of on-farm interventions by dairy farmers. While the use of vaccines or dietary supplements may decrease STEC O157 bacterial shedding in cattle (36), there is currently limited economic incentive for New Zealand dairy farmers to allocate time and money to prevent a bacterium from colonizing what are considered “surplus,” relatively low-value animals with no clinical signs. STEC and other E. coli bacteria are considered part of the normal bovine microbiota; therefore elimination of STEC from a herd and farm environment may be an unrealistic goal. Previously validated on-farm intervention strategies that are easily adopted and cost-effective and which target mutual critical control points for several pathogens (e.g., STEC, Campylobacter, Salmonella, and Cryptosporidium) could form the basis of multiple-agent control methods to reduce the overall level of zoonoses. This could impact overall STEC prevalence in animals and reduce the likelihood of human infection. Given that STEC bacteria are found in cattle throughout the world, focusing on methods to decrease human exposure by minimizing the presence of STEC in food and minimizing environmental exposure is likely to be more beneficial than attempting to eliminate the presence of STEC in ruminant reservoirs.

The findings of this study provide important baseline data regarding the national prevalence of a zoonotic pathogen on New Zealand dairy farms. Future goals for STEC research should be multimodal, addressing issues that could benefit the meat industry and protect public health by using social science, epidemiology, and molecular biology.

## MATERIALS AND METHODS

The Animal Ethics Committee of Massey University, Palmerston North, New Zealand approved this study on 17 April 2014, under protocol number 14/29.

### Sample size calculations.

We performed sample size calculations using a cluster-sample calculation with a design effect of 3.6, based on a previous repeated cross-sectional study of STEC O26 and STEC O157 at cattle processing plants in New Zealand (16). Table S5 in the supplemental material contains sample size calculations for the numbers of farms and calves required to be 95% certain that the prevalence estimate is within ±20% of the true prevalence. Given previous estimates of STEC O26 and STEC O157 prevalence in calves in New Zealand, we used a conservative estimate of 20% farm prevalence of the “Top 7” STEC, and we aimed to recruit a minimum of 93 farms and sample a maximum of 15 calves per farm. The critical probability for all statistical analyses was *P* < 0.05.

### Random stratified farm selection.

We selected farms using a stratified random sampling scheme based on regionally proportioned sampling of the number of farms in each region. We targeted the six largest dairy regions, which account for approximately 75% of the dairy farms in New Zealand, namely, Northland, Waikato, Taranaki, Manawatu-Wellington, Canterbury, and Southland (37). Given a 60-day calving period, only farms with a documented herd size of more than 150 milking cows were eligible, to ensure enough calves would be present on the day of sampling. Potential farms were selected randomly from a national farm database (Agribase; AsureQuality Ltd., Auckland, New Zealand) and contacted by telephone, leading to the recruitment of 102 dairy farms (see Table S6 in the supplemental material).

### Random animal selection and sampling within calf pens on farms.

We categorized calves into two groups: young calves from 2 to 9 days of age and older calves from 10 to 21 days of age. Given the focus on “Top 7” STEC prevalence in very young calves, where possible, 10 calves were sampled in the young age group and 5 in the older age group.

Sampling of calves occurred during a single farm visit from 28 July to 24 September during the 2014 spring calving season. For this study, a pen was defined as an enclosed area where calves had direct contact with each other and shared water and feeding resources. After identifying calf ages, up to three pens were selected that allowed for the maximum number of animals in the two age groups to be sampled, with equal numbers per pen where possible. A random number generator was used to select pens if more than three suitable pens were available for sampling. If more than five animals were present in a pen, a spin-pointer mobile phone application was used to randomly select the first calf to be sampled, after which animals were chosen in an alternating manner in the clockwise direction, in proportion to the total calves in the pen. Calves were marked for selection and then again following sampling to maintain the random selection and prevent resampling.

Animals were excluded from sampling if they appeared to be injured or sick, based on visual clinical assessment by the sampler (A.S. Browne, a registered veterinarian). In total, 1,508 young calves from 267 pens were sampled by collecting recto-anal mucosal swabs (RAMS) from each calf using Amies transport swabs (Copan Diagnostics Inc., Brescia, Italy). All RAMS were kept on ice in an insulated container immediately after sampling and shipped for processing the same day as they were collected.

### Initial laboratory processing.

All RAMS were shipped on ice overnight to ^*m*^EpiLab, Massey University, Palmerston North, and enriched in modified tryptone soya broth (mTSB; Oxoid Limited, Hampshire, United Kingdom) at 42°C for 15 to 21 h. Genomic DNA was extracted from 1 ml of enrichment broth using a double-wash boil preparation method, according to the GeneSeek laboratory's instructions, and frozen at −80°C. The DNA samples were shipped to GeneSeek Operations (Lincoln, NE) on dry ice. All samples were analyzed using the PCR/MALDI-TOF assay NeoSEEK (NeoSEEK STEC confirmation; Neogen Corporation, Lansing, MI), for presence of “Top 7” STEC.

### Evaluation of NeoSEEK for New Zealand “Top 7” STEC detection.

NeoSEEK uses PCR amplification to generate allele-specific DNA products of different masses and chip-based mass spectrometry to analyze the extension products. The assay is based on the presence of single nucleotide polymorphisms (SNPs) in the O-antigen gene cluster that can differentiate between STEC and non-STEC bacterial strains of the same serogroup (38), as well as other targets (i.e., virulence genes). NeoSEEK uses over 89 gene targets via PCR/MALDI-TOF to detect the presence of the “Top 7” STEC without the need for agar-based culture isolation (E. Hosking, personal communication). This assay has a letter of no objection from the U.S. Department of Agriculture Food Safety and Inspection Service (USDA-FSIS), and is used commercially as a confirmation method for detection of STEC in ground beef and beef trim. As far as we are aware, the evaluation and application of this technology in this study to detect fecal carriage of STEC in calves is unique.

A technical report, including summary data from the study conducted for NeoSEEK to receive a letter of no objection, is available online (39). Prior to field collection of samples for this study, 100 characterized New Zealand STEC and non-STEC isolates from six serogroups (O26, O45, O103, O121, O145, and O157; *n* = 88), as well as from non-“Top 7” serogroups (*n* = 12), from the Ministry of Primary Industries (*n* = 64) and the Hopkirk Institute (*n* = 36) culture collections were obtained and used by the Institute of Environmental Science and Research to evaluate the detection efficacy of the NeoSEEK assay. One Australian STEC O111 isolate was also tested, as no STEC O111 had been isolated in New Zealand. All 101 isolates had undergone serological analysis and previously been characterized by PCR for the presence of *stx*_1_, *stx*_2_, and *eae* virulence markers; there was 100% concordance with the NeoSEEK assay.

All DNA samples derived from the calf fecal samples, in addition to being submitted for NeoSEEK analysis, were tested for the “Top 7” serogroups at ^*m*^EpiLab, using a real-time PCR (RT-PCR) assay (40). All DNA samples were run with positive, negative, and blank template controls using PerfeCTa Multiplex quantitative PCR (qPCR) ToughMix (Quanta Biosciences, Beverly, MA) on a Rotor-Gene Q 5plex high-resolution melting (HRM) platform (Qiagen, Hilden, Germany). In-house validation of the RT-PCR method revealed a limit of detection (LOD) of 10^2^ CFU per ml for all serogroups evaluated, except for O157 and O103, for which the LOD was 10^1^. The LOD of the NeoSEEK assay was approximately 10^3^ CFU/ml (E. Hosking, personal communication).

**FIG 7:**
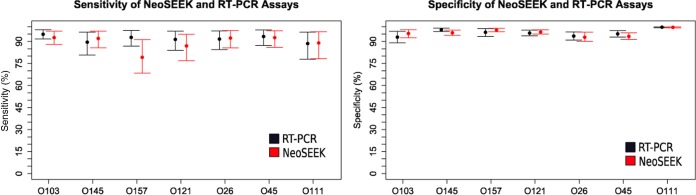
Sensitivity and specificity of NeoSEEK and real-time PCR assays for detection of the “Top 7” serogroups in calf fecal enrichment samples (*n* = 1,508).

Latent class modeling (41) was used to estimate the sensitivity and specificity of serogroup detection of the “Top 7” STEC serogroups. This modeling technique is used to compare two diagnostic tests when neither is considered a gold standard. Latent class analyses were performed (https://github.com/jmarshallnz/lcar) to calculate a 95% CI for the sensitivity and specificity of the NeoSEEK and RT-PCR methods for detection of all seven serogroups for the 1,508 DNA samples (Fig. 7; see also Tables S7 and S8 in the supplemental material). Latent class analyses also produced prevalence estimates of all seven serogroups by three factors, namely, region (*n* = 6), age of calf (young and old), and location in the North or South Island.

All 1,508 calf RAMS samples collected were enriched and stored in a glycerol (4:1 ratio) suspension in a −80°C freezer. The isolation of individual STEC colonies from frozen enrichment broth was important for confirming the STEC detection using molecular methods (NeoSEEK, RT-PCR), as well as analysis of the bacterial isolates using whole-genome sequencing. Recovery of bacteria from frozen enrichment broth samples was attempted, based on the NeoSEEK assay results for “Top 7” STEC-positive samples.

Due to the large number of STEC detections by analysis of all 1,508 samples using the NeoSEEK assay (*n* = 408) and the costs and labor required for testing and isolation of bacteria from enrichment broth samples, isolation was prioritized based on serogroup. Due to their public health importance, recovery was attempted on all STEC O157-positive (*n* = 29) and STEC O26-positive (*n* = 109) samples, using a modification of USDA-FSIS methods (42, 43). STEC recovery was attempted on samples using sorbitol MacConkey agar supplemented with cefixime and tellurite (CT-SMAC) and rhamnose MacConkey agar supplemented with cefixime and tellurite (CT-RMAC) (Fort Richard Laboratories, Auckland, New Zealand) for STEC O157 and STEC O26, respectively, using immunomagnetic separation beads (IMS) (Abraxis, Warminister, PA). The methods used were adapted from the USDA-FSIS methods (42, 43) to include an initial “direct” culture screen, where frozen glycerol enrichment culture was plated directly onto selective agar (CT-SMAC for O157 and CT-RMAC for O26). If target STEC serogroups were not identified, frozen glycerol enrichment broth was reenriched in mTSB broth, and immunomagnetic separation (IMS) was attempted according to the manufacturer's instructions. Up to 10 colonies were tested for the specific serogroups on a plate using latex agglutination, and up to four positive individual isolates were subcultured and stored frozen with glycerol. Subcultured isolates were confirmed for serogroup and tested for virulence-associated genes using an in-house RT-PCR (40).

### Whole-genome sequencing, assembly, and analysis of E. coli serogroup O26 isolates retrieved from calf fecal samples.

We used random stratified selection by region, farm, and calf pen to select 66 serogroup O26 bacterial isolates (45/66 STEC O26 and 21/66 nontoxigenic O26) for whole-genome sequencing. Multiple isolates were selected from four calves to evaluate within-animal diversity. We performed DNA extraction from a single colony picked from Columbia horse blood agar (Fort Richard Laboratories, Auckland, New Zealand) using the QIAamp DNA minikit (Qiagen, Hilden, Germany) and prepared the libraries using the Nextera XT DNA library preparation kit (Illumina, San Diego, CA). Prepared libraries were submitted to New Zealand Genomics Limited (University of Otago, Dunedin, New Zealand), which performed sequencing using Illumina MiSeq 2 × 250-bp paired-end (PE) and Illumina HiSeq 2 × 125-bp PE v4 instruments. Processed reads are publicly available on the NCBI Sequence Read Archive (SRA) under BioProject no. PRJNA396667, and Table S9 in the supplemental material lists the metadata and accession numbers of the sequences.

Raw sequences were evaluated, assembled, annotated, and analyzed using the Nullarbor pipeline in “accurate” mode (21). RaxML maximum-likelihood trees were generated from Roary data for core genes via single nucleotide polymorphism (SNP) analysis of core genes and for accessory genes via a presence/absence matrix without an external reference (44). Assembled genomes were batch uploaded to the Center for Genomic Epidemiology server for identification of virulence factors, multilocus sequence type (ST), antimicrobial resistance genes, and somatic (O) and flagellar (H) type (22). A distance matrix was created from the SNP distances between isolates, and a dissimilarity matrix was created from the presence/absence matrix of the accessory genome from Roary, as well as the 26 virulence genes predicted by the Center for Genomic Epidemiology output, and all three were evaluated using the PERMANOVA and CLUSTER packages (PRIMER-E; Quest Research Limited, Auckland, New Zealand), with region and farm as independent factors.

Figures depicting phylogenetic relationships and associated variables were created using Interactive Tree of Life (iTOL) software (45) and further amended using Inkscape open source software version 0.92.2 (https://inkscape.org).

### Data retrieval and statistical analysis.

At the time of the visit, written consent to participate in the study was obtained from a manager on every farm. Animal-and farm-level data, including management and environmental factors, were collected from each farm through observation, electronic devices, and interviewing a manager on every farm (see Table S10 in the supplemental material).

All statistical analyses were performed using R version 3.2.1 (46). Eight outcome variables were considered, consisting of the presence or absence of each of the “Top 7” STEC bacteria, and an additional variable specifying the presence or absence of any of the “Top 7” STEC bacteria. All factors were first assessed using machine learning techniques from the randomForest package (47). The most important 10% of factors identified in the randomForest analysis were considered explanatory fixed effects in a linear mixed-effects model, with “pen” within “farm” included as random effects variables. A preliminary model was generated by stepwise backward elimination of the least significant variables, and eliminated variables were assessed for confounding. Confounding variables, determined by a change of >30% in the main variable coefficient, were kept in the model, even if they were nonsignificant. The intraclass correlation coefficient (ρ) was calculated using the iccbin function in the aod package with a Monte Carlo 1-way generalized linear mixed model (48). A description of the strength of correlation is as follows: 0.00 to 0.19, very weak; 0.20 to 0.39, weak; 0.40 to 0.59, moderate; 0.60 to 0.79, strong; and 0.80 to 1.0, very strong.

### Accession number(s).

Processed reads are publicly available in the NCBI Sequence Read Archive (SRA) under BioProject number PRJNA396667 and BioSample accession numbers SAMN07430764 to SAMN07430774, SAMN07430783 to SAMN07430788, SAMN07430810 to SAMN07430825, SAMN07430840 to SAMN07430853, and SAMN07430875 to SAMN07430893. See Table S9 in the supplemental material for other related metadata.

## Supplementary Material

Supplemental material
