# Supplementary material for: Molecular Epidemiology of Shiga Toxin-Producing Escherichia coli (STEC) on New Zealand Dairy Farms: Application of a Culture-Independent Assay and Whole-Genome Sequencing
Source: Appl Environ Microbiol. 2018 Jul 2;84(14):e00481-18. doi: 10.1128/AEM.00481-18 (PMC6029106; doi:10.1128/AEM.00481-18)
Supplement: Supplemental material [file supp_84_14_e00481-18__index.html]

Supplemental material 

# Molecular Epidemiology of Shiga Toxin-Producing Escherichia coli (STEC) on New Zealand Dairy Farms: Application of a Culture-Independent Assay and Whole-Genome Sequencing

## Supplemental material

- Supplemental file 1 -

  Detection of “Top 7” STEC in calves on farms (Table S1); logistic mixed-effects regression model of factors associated with prevalence of any STEC O26 (Table S2), STEC O103 (Table S3), or STEC O145 (Table S4); sample size calculations for farms and calves with cluster-sample design effect (Table S5); New Zealand dairy farms per region sampled and milking herd size (Table S6); sensitivity (Table S7) and specificity (Table S8) of NeoSEEK and reverse transcriptase PCR assays for detection of “Top 7” STEC serogroups in calf fecal enrichment samples; BioProject PRJNA396667 metadata from raw sequence data used in this study (Table S9); location, animal, pen, and farm management factors evaluated against outcome variables of “Top 7” STEC prevalence in dairy calves (Table S10); heatmap of SNP distances between *E. coli* serogroup O26 isolates (Fig. S1).

  PDF, 307K
