# Supplementary material for: Molecular Epidemiology of Shiga Toxin-Producing Escherichia coli (STEC) on New Zealand Dairy Farms: Application of a Culture-Independent Assay and Whole-Genome Sequencing
Source: Appl Environ Microbiol. 2018 Jul 2;84(14):e00481-18. doi: 10.1128/AEM.00481-18 (PMC6029106; doi:10.1128/AEM.00481-18)
Supplement: Supplemental material [file AEM.00481-18_zam014188630s1.pdf]

Supplemental Materials

Table S1: Detection of 'Top 7' STEC in calves (n=1508) on farms (n=102) by number of 'Top 7' STEC serogroups detected

Farm ID	Calves with no 'Top 7' STEC detected	Calves with one 'Top 7' STEC detected	Calves with two 'Top 7' STEC detected	Calves with three 'Top 7' STEC detected
VCF1	15			
VCF2	15			
VCF3	14	1		
VCF4	10	4	1	
VCF5	13	2		
VCF6	12		3	
VCF7	13	2		
VCF8	14	1		
VCF9	14	1		
VCF10	15			
VCF11	13	2		
VCF12	15			
VCF13	13	2		
VCF14	14	1		
VCF15	9	5	1	
VCF16	4	10	1	
VCF17	12	3		
VCF18	8	4	3	
VCF19	12	2	1	
VCF20	1	9	4	1
VCF21		3	7	5
VCF22	11	2	1	1
VCF23	15			
VCF24	15			
VCF25	15			
VCF26	10	3	2	
VCF27	3	10	2	

VCF28	13	1	1	
VCF29	14	1		
VCF30	14	1		
VCF31	9	6		
VCF32	8	3	3	1
VCF33	15			
VCF34	11	3	1	
VCF35	9	3	3	
VCF36	13	2		
VCF37	8	2	4	1
VCF38	13	2		
VCF39	12	3		
VCF40	15			
VCF41	15			
VCF42	14	1		
VCF43	10	1	1	
VCF44	15			
VCF45	15			
VCF46	15			
VCF47	11	3	1	
VCF48	12	3		
VCF49	15			
VCF50	15			
VCF51	13	2		
VCF52	14		1	
VCF53	14	1		
VCF54	13	2		
VCF55	13	2		
VCF56	5	5	3	2
VCF57	8	2	5	
VCF58	10	4	1	
VCF59	9	4	2	
VCF60	15			
VCF61	14	1		
VCF62	15			
VCF63	9	4		

VCF64	5	4	6	
VCF65	14	1		
VCF66	15			
VCF67	8			
VCF68	14	1		
VCF69	13	2		
VCF70	15			
VCF71	7	8		
VCF72	11	4		
VCF73	15			
VCF74	15			
VCF75	13	2		
VCF76	11	2	1	
VCF77	11	3	1	
VCF78	15			
VCF79	15			
VCF80	11	2	2	
VCF81	9	6		
VCF82	14	1		
VCF83	14	1		
VCF84	12	3		
VCF85	11	4		
VCF86	6			
VCF87	11	4		
VCF88	13	2		
VCF89	9	3	2	1
VCF90	13	1	1	
VCF91	13	1		1
VCF92	15			
VCF93	7	6	2	
VCF94	12	3		
VCF95	11	2	2	
VCF96	8	4	3	
VCF97	13		2	
VCF98	14		1	
VCF99	11	4		

VCF100	7	7	1	
VCF101	12	3		
VCF102	11	4		
Totals	1202	217	76	13

Table S2: Logistic mixed effects regression model of factors associated with prevalence of any STEC O26

Factor	OR	95% CI	p-value
**Number of calves** in calf pen (increase of 1 calf)	1.07	1.02, 1.13	0.012*

*Significant variable (p<0.05)

Random Effects Variance: Pen within Farm (Variance = 3.26), Farm (Variance = 33.36)

Table S3: Logistic mixed effects regression model of factors associated with prevalence of any STEC O103

Factor	OR	95% CI	p-value
**Humidity**: Inside calf pen vs. outside the calf housing area (increase of 1% relative humidity)	1.02	1.01, 1.03	0.002*
**High ammonia in calf pen** (subjective measurement)	1.17	1.00, 1.36	0.047*

*Significant variable (p<0.05)

Random Effects Variance: Pen within Farm (Variance = 4.49), Farm (Variance = 0.90)

Table S4: Logistic mixed effects regression model of factors associated with prevalence of any STEC O145

Factor	OR	95% CI	p-value
**Region** (compared to Canterbury)			0.002#
Northland	1.38	1.15, 1.66	<0.001*
Waikato	0.98	0.84, 1.14	0.805
Taranaki	1.01	0.85, 1.20	0.886
Manawatu-Wellington	1.03	0.86, 1.24	0.742
Southland	1.02	0.85, 1.23	0.811
**Number of calves** in calf pen (increase of 1 calf)	1.04	0.999, 1.08	0.059†
**Age**: Young calves (2 to 9 days of age) vs. older calves (10 to 21 days of age)	0.89	0.84, 0.95	<0.001*

*Significant variable (p<0.05)

#Likelihood-ratio test of variable as a whole

†Factor left in model as it approaches significant value

Random Effects Variance: Pen within farm (Variance = 1.56), Farm (Variance = 2.69)

Table S5: Sample size calculations for farms and calves with cluster-sample design effect (rho=3.6)

**Estimated 'Top 7' STEC farm prevalence***	**Farms needed**	**Calves per farm**	**Total samples**
25%	53	20	1060
25%	70	15	1050
25%	105	10	1050
20%	70	20	1400
20%	93	15	1395
20%	139	10	1390
15%	99	20	1980
15%	132	15	1980
15%	197	10	1970

*Based on one animal being positive for any of the Top 7 STEC

Table S6: New Zealand dairy farms per region sampled and milking herd size, based on farm manager records

**Region** (number of farms)	**Median herd size**	**Range***
Northland (10)	210	35 to 920
Waikato (35)	360	120 to 960
Taranaki (19)	320	140 to 560
Manawatu-Wellington (12)	440	320 to 1100
Canterbury (14)	800	200 to 1960
Southland (12)	580	440 to 980

*Farms in three regions contained fewer than the target 150 milking herd size on the day of sampling; adequate numbers of calves were sampled

Table S7: Sensitivity of NeoSEEK and reverse transcriptase PCR assays for detection of Top 7 STEC serogroups in calf fecal enrichment samples (n=1,508)

Serogroup	Sensitivity RT-PCR		Sensitivity NeoSEEK	
	Mean	95% credible interval	Mean	95% credible interval
O103	0.95	0.91-0.97	0.93	0.88-0.97
O145	0.89	0.81-0.96	0.92	0.85-0.97
O157	0.93	0.87-0.98	0.79	0.69-0.90
O121	0.91	0.84-0.97	0.87	0.77-0.95
O26	0.91	0.84-0.97	0.92	0.85-0.97
O45	0.93	0.87-0.98	0.93	0.87-0.97
O111	0.89	0.77-0.96	0.89	0.89-0.96

Table S8: Specificity of NeoSEEK and reverse transcriptase PCR assays for detection of Top 7 STEC serogroups in calf fecal enrichment samples (n=1,508)

	**Specificity RT-PCR**		**Specificity NeoSEEK**	
**Serogroup**	**Mean**	**95% CI**	**Mean**	**95% CI**
O103	0.93	0.89-0.97	0.95	0.93-0.98
O145	0.98	0.97-0.99	0.96	0.94-0.98
O157	0.96	0.94-0.99	0.98	0.97-0.99
O121	0.96	0.94-0.98	0.96	0.95-0.98
O26	0.94	0.91-0.97	0.93	0.90-0.96
O45	0.95	0.93-0.97	0.93	0.91-0.96
O111	1.00	0.99-1.00	1.00	0.99-1.00

Table S9: BioProject PRJNA396667 metadata from raw sequence data used in this study, publicly available on the NCBI SRA archive

BioSample accession	sample_ name	collection_ date	geo_loc_ name	Isolati on_ source	lat_lon	host	host _age	host	host_disease	serotype
SAMN07430764	VC1139e	11-Sep-14	New Zealand: Canterbury	bovine	43.75 S 171.16 E	Bos taurus taurus	Calf	VC1139	Reservoir	O26:H11
SAMN07430765	VC1140e	11-Sep-14	New Zealand: Canterbury	bovine	43.75 S 171.16 E	Bos taurus taurus	Calf	VC1140	Reservoir	O26:H11
SAMN07430766	VC1186e	15-Sep-14	New Zealand: Canterbury	bovine	43.75 S 171.16 E	Bos taurus taurus	Calf	VC1186	Reservoir	O26:H11
SAMN07430767	VC1187e	15-Sep-14	New Zealand: Canterbury	bovine	43.75 S 171.16 E	Bos taurus taurus	Calf	VC1187	Reservoir	O26:H11
SAMN07430768	VC1190e	15-Sep-14	New Zealand: Canterbury	bovine	43.75 S 171.16 E	Bos taurus taurus	Calf	VC1190	Reservoir	O26:H11
SAMN07430769	VC1195e	15-Sep-14	New Zealand: Canterbury	bovine	43.75 S 171.16 E	Bos taurus taurus	Calf	VC1195	Reservoir	O26:H11
SAMN07430770	VC1196e	15-Sep-14	New Zealand: Canterbury	bovine	43.75 S 171.16 E	Bos taurus taurus	Calf	VC1196	Reservoir	O26:H11
SAMN07430771	VC1202e	15-Sep-14	New Zealand: Canterbury	bovine	43.75 S 171.16 E	Bos taurus taurus	Calf	VC1202	Reservoir	O26:H11
SAMN07430772	VC1309e	17-Sep-14	New Zealand: Canterbury	bovine	43.75 S 171.16 E	Bos taurus taurus	Calf	VC1309	Reservoir	O26:H11
SAMN07430773	VC1310e	17-Sep-14	New Zealand: Canterbury	bovine	43.75 S 171.16 E	Bos taurus taurus	Calf	VC1310	Reservoir	O26:H11
SAMN07430774	VC1311e	17-Sep-14	New Zealand: Canterbury	bovine	43.75 S 171.16 E	Bos taurus taurus	Calf	VC1311	Reservoir	O26:H11
SAMN07430783	VC833e	27-Aug-14	New Zealand: Manawatu-Wellington	bovine	39.73 S 175.44 E	Bos taurus taurus	Calf	VC833	Reservoir	O26:H11
SAMN07430784	VC833f	27-Aug-14	New Zealand: Manawatu-Wellington	bovine	39.73 S 175.44 E	Bos taurus taurus	Calf	VC833	Reservoir	O26:H11
SAMN07430785	VC833g	27-Aug-14	New Zealand: Manawatu-Wellington	bovine	39.73 S 175.44 E	Bos taurus taurus	Calf	VC833	Reservoir	O26:H11
SAMN07430786	VC833h	27-Aug-14	New Zealand: Manawatu-Wellington	bovine	39.73 S 175.44 E	Bos taurus taurus	Calf	VC833	Reservoir	O26:H11

SAMN07430787	VC836e	27-Aug-14	New Zealand: Manawatu-Wellington	bovine	39.73 S 175.44 E	Bos taurus taurus	Calf	VC836	Reservoir	O26:H11
SAMN07430788	VC837e	27-Aug-14	New Zealand: Manawatu-Wellington	bovine	39.73 S 175.44 E	Bos taurus taurus	Calf	VC837	Reservoir	O26:H11
SAMN07430810	VC1362e	22-Sep-14	New Zealand: Southland	bovine	45.85 S 167.68 E	Bos taurus taurus	Calf	VC1362	Reservoir	O26:H11
SAMN07430811	VC1366e	22-Sep-14	New Zealand: Southland	bovine	45.85 S 167.68 E	Bos taurus taurus	Calf	VC1366	Reservoir	O26:H11
SAMN07430812	VC1367e	22-Sep-14	New Zealand: Southland	bovine	45.85 S 167.68 E	Bos taurus taurus	Calf	VC1367	Reservoir	O26:H11
SAMN07430813	VC1394e	23-Sep-14	New Zealand: Southland	bovine	45.85 S 167.68 E	Bos taurus taurus	Calf	VC1394	Reservoir	O26:H11
SAMN07430814	VC1395e	23-Sep-14	New Zealand: Southland	bovine	45.85 S 167.68 E	Bos taurus taurus	Calf	VC1395	Reservoir	O26:H11
SAMN07430815	VC1395f	23-Sep-14	New Zealand: Southland	bovine	45.85 S 167.68 E	Bos taurus taurus	Calf	VC1395	Reservoir	O26:H11
SAMN07430816	VC1395g	23-Sep-14	New Zealand: Southland	bovine	45.85 S 167.68 E	Bos taurus taurus	Calf	VC1395	Reservoir	O26:H11
SAMN07430817	VC1396e	23-Sep-14	New Zealand: Southland	bovine	45.85 S 167.68 E	Bos taurus taurus	Calf	VC1396	Reservoir	O26:H11
SAMN07430818	VC1403e	23-Sep-14	New Zealand: Southland	bovine	45.85 S 167.68 E	Bos taurus taurus	Calf	VC1403	Reservoir	O26:H11
SAMN07430819	VC1471e	24-Sep-14	New Zealand: Southland	bovine	45.85 S 167.68 E	Bos taurus taurus	Calf	VC1471	Reservoir	O26:H11
SAMN07430820	VC1471f	24-Sep-14	New Zealand: Southland	bovine	45.85 S 167.68 E	Bos taurus taurus	Calf	VC1471	Reservoir	O26:H11
SAMN07430821	VC1471g	24-Sep-14	New Zealand: Southland	bovine	45.85 S 167.68 E	Bos taurus taurus	Calf	VC1471	Reservoir	O26:H11
SAMN07430822	VC1471h	24-Sep-14	New Zealand: Southland	bovine	45.85 S 167.68 E	Bos taurus taurus	Calf	VC1471	Reservoir	O26:H11
SAMN07430823	VC1473e	24-Sep-14	New Zealand: Southland	bovine	45.85 S 167.68 E	Bos taurus taurus	Calf	VC1473	Reservoir	O26:H11
SAMN07430824	VC1474e	24-Sep-14	New Zealand: Southland	bovine	45.85 S 167.68 E	Bos taurus taurus	Calf	VC1474	Reservoir	O26:H11
SAMN07430825	VC1486e	24-Sep-14	New Zealand: Southland	bovine	45.85 S 167.68 E	Bos taurus taurus	Calf	VC1486	Reservoir	O26:H11

SAMN07430840	VC1113e	4-Sep-14	New Zealand: Taranaki	bovine	39.35 S 174.44 E	Bos taurus taurus	Calf	VC1113	Reservoir	O26:H11
SAMN07430841	VC1122f	4-Sep-14	New Zealand: Taranaki	bovine	39.35 S 174.44 E	Bos taurus taurus	Calf	VC1122	Reservoir	O26:H11
SAMN07430842	VC1125e	4-Sep-14	New Zealand: Taranaki	bovine	39.35 S 174.44 E	Bos taurus taurus	Calf	VC1125	Reservoir	O26:H11
SAMN07430843	VC880e	1-Sep-14	New Zealand: Taranaki	bovine	39.35 S 174.44 E	Bos taurus taurus	Calf	VC880	Reservoir	O26:H11
SAMN07430844	VC932f	1-Sep-14	New Zealand: Taranaki	bovine	39.35 S 174.44 E	Bos taurus taurus	Calf	VC932	Reservoir	O26:H11
SAMN07430845	VC936e	1-Sep-14	New Zealand: Taranaki	bovine	39.35 S 174.44 E	Bos taurus taurus	Calf	VC936	Reservoir	O26:H11
SAMN07430846	VC940e	1-Sep-14	New Zealand: Taranaki	bovine	39.35 S 174.44 E	Bos taurus taurus	Calf	VC940	Reservoir	O26:H11
SAMN07430847	VC943e	2-Sep-14	New Zealand: Taranaki	bovine	39.35 S 174.44 E	Bos taurus taurus	Calf	VC943	Reservoir	O26:H11
SAMN07430848	VC943f	2-Sep-14	New Zealand: Taranaki	bovine	39.35 S 174.44 E	Bos taurus taurus	Calf	VC943	Reservoir	O26:H11
SAMN07430849	VC943g	2-Sep-14	New Zealand: Taranaki	bovine	39.35 S 174.44 E	Bos taurus taurus	Calf	VC943	Reservoir	O26:H11
SAMN07430850	VC943h	2-Sep-14	New Zealand: Taranaki	bovine	39.35 S 174.44 E	Bos taurus taurus	Calf	VC943	Reservoir	O26:H11
SAMN07430851	VC946e	2-Sep-14	New Zealand: Taranaki	bovine	39.35 S 174.44 E	Bos taurus taurus	Calf	VC946	Reservoir	O26:H11
SAMN07430852	VC951e	2-Sep-14	New Zealand: Taranaki	bovine	39.35 S 174.44 E	Bos taurus taurus	Calf	VC951	Reservoir	O26:H11
SAMN07430853	VC955e	2-Sep-14	New Zealand: Taranaki	bovine	39.35 S 174.44 E	Bos taurus taurus	Calf	VC955	Reservoir	O26:H11
SAMN07430875	VC396e	12-Aug-14	New Zealand: Waikato	bovine	38.06 S 175.44 E	Bos taurus taurus	Calf	VC396	Reservoir	O26:H11
SAMN07430876	VC397e	12-Aug-14	New Zealand: Waikato	bovine	38.06 S 175.44 E	Bos taurus taurus	Calf	VC397	Reservoir	O26:H11
SAMN07430877	VC401e	12-Aug-14	New Zealand: Waikato	bovine	38.06 S 175.44 E	Bos taurus taurus	Calf	VC401	Reservoir	O26:H11
SAMN07430878	VC452e	13-Aug-14	New Zealand: Waikato	bovine	38.06 S 175.44 E	Bos taurus taurus	Calf	VC452	Reservoir	O26:H11

SAMN07430879	VC456e	13-Aug-14	New Zealand: Waikato	bovine	38.06 S 175.44 E	Bos taurus taurus	Calf	VC456	Reservoir	O26:H11
SAMN07430880	VC459e	13-Aug-14	New Zealand: Waikato	bovine	38.06 S 175.44 E	Bos taurus taurus	Calf	VC459	Reservoir	O26:H11
SAMN07430881	VC473e	13-Aug-14	New Zealand: Waikato	bovine	38.06 S 175.44 E	Bos taurus taurus	Calf	VC473	Reservoir	O26:H11
SAMN07430882	VC474e	13-Aug-14	New Zealand: Waikato	bovine	38.06 S 175.44 E	Bos taurus taurus	Calf	VC474	Reservoir	O26:H11
SAMN07430883	VC474f	13-Aug-14	New Zealand: Waikato	bovine	38.06 S 175.44 E	Bos taurus taurus	Calf	VC474	Reservoir	O26:H11
SAMN07430884	VC474g	13-Aug-14	New Zealand: Waikato	bovine	38.06 S 175.44 E	Bos taurus taurus	Calf	VC474	Reservoir	O26:H11
SAMN07430885	VC474h	13-Aug-14	New Zealand: Waikato	bovine	38.06 S 175.44 E	Bos taurus taurus	Calf	VC474	Reservoir	O26:H11
SAMN07430886	VC476e	13-Aug-14	New Zealand: Waikato	bovine	38.06 S 175.44 E	Bos taurus taurus	Calf	VC476	Reservoir	O26:H11
SAMN07430887	VC477e	13-Aug-14	New Zealand: Waikato	bovine	38.06 S 175.44 E	Bos taurus taurus	Calf	VC477	Reservoir	O26:H11
SAMN07430888	VC479e	13-Aug-14	New Zealand: Waikato	bovine	38.06 S 175.44 E	Bos taurus taurus	Calf	VC479	Reservoir	O26:H11
SAMN07430889	VC545e	18-Aug-14	New Zealand: Waikato	bovine	38.06 S 175.44 E	Bos taurus taurus	Calf	VC545	Reservoir	O26:H11
SAMN07430890	VC547e	18-Aug-14	New Zealand: Waikato	bovine	38.06 S 175.44 E	Bos taurus taurus	Calf	VC547	Reservoir	O26:H11
SAMN07430891	VC550e	18-Aug-14	New Zealand: Waikato	bovine	38.06 S 175.44 E	Bos taurus taurus	Calf	VC550	Reservoir	O26:H11
SAMN07430892	VC554e	18-Aug-14	New Zealand: Waikato	bovine	38.06 S 175.44 E	Bos taurus taurus	Calf	VC554	Reservoir	O26:H11
SAMN07430893	VC555e	18-Aug-14	New Zealand: Waikato	bovine	38.06 S 175.44 E	Bos taurus taurus	Calf	VC555	Reservoir	O26:H11

Table S10: Location, animal, pen, and farm management factors evaluated against outcome variables of the Top 7 STEC prevalence in dairy calves

Risk Factor Level	Variable	Type	Notes	Source of measurement
Location	Region	Categorical	6 regions	
	Elevation	Continuous	Meters	GPS device
	Humidity	Continuous	Measured in open space no less than 5 meters from Pen	Thermo-Hygrometer
	Temperature	Continuous	Measured in open space no less than 5 meters from Pen	Thermo-Hygrometer
**Animal**	Age	Binary	Young (2 to 9 days); Old (10 to 21 days)	
	Sex	Binary	Male, Female	
	Breed	Categorical		
	Class	Categorical	Bobby, Beef, Replacement	
	FecalScore	Ordinal	Coverage of hide with fecal matter or mud: 1 (0-25%), 2 (25-50%), 3 (50-75%), 4 (75-100%)	Visual assessment of fecal contamination on hide
**Calf Pen**	PenAnimalCount	Continuous	Number of calves in pen	
	PenOrientation	Categorical	Primary direction that pen faced; 8 cardinal points	GPS device
	PenHumidity	Continuous	Recorded in centre of pen	Thermo-hygrometer
	PenTemperature	Continuous	Recorded in centre of pen	Thermo-hygrometer
	PenType	Categorical	Open, closed, or conversion	
	Floor	Categorical	Flooring beneath substrate	
	Substrate	Categorical	Type of substrate used as bedding for calves	

	SubstrateCleaned	Binary	Whether substrate was cleaned during the calving season	
	SubstrateCleanedFreq	Continuous	How often pen substrate was cleaned	
	SubstrateTopped	Binary	Was new pen substrate added during the season	
	SubstrateToppedFreq	Continuous	How often new substrate was added	
	SubstrateSprayed	Binary	Was the pen sprayed with disinfectant during the season	
	SubstrateSprayedFreq	Continuous	How often pen was sprayed	
	PenFecalContamination	Ordinal	4 Groups: 0-25%, 25-50%, 50-75%, 75-100%	Visual assessment of fecal matter on pen floor
	PenAmmonia	Binary	Irritation of mucous membranes (nasal passage, eyes) to sampler (ASB)	
	TempInsideOutside	Continuous	Temperature inside pen minus temperature outside the calf housing area (~10 meters from the building)	
	HumidityInsideOutside	Continuous	Humidity inside pen minus humidity outside calf housing area (~10 meters from the building)	
**Farm Management**	MilkingHerdSize	Continuous	Number of cows in milking herd	
	SpringCalvingSize	Continuous	Number of cows in spring calving herd	
	DairyOnly	Binary	Only dairy cattle raised	
	BeefDairy	Binary	Beef and dairy cattle raised	

	MeatCompany	Categorical	Meat company used for veal calf processing	
	Pigs	Binary	Presence of pigs on farm	
	Deer	Binary	Presence of deer on farm	
	Sheep	Binary	Presence of sheep on farm	
	Goats	Binary	Presence of goats on farm	
	Horses	Binary	Presence of horses on farm	
	CalvesOtherFarms	Binary	Importation of calves from other farms in past two calving seasons	
	CowsOtherFarms	Binary	Importation of cows from other farms in the past two calving seasons	
	CalvingDateSpring	Date	Planned start of winter calving	
	CalvingDateAutumn	Date	Planned start of autumn calving (if applicable)	
	DaysSinceStartofCalving	Continuous	Days from start of calving to day of sampling for study	
	YearRoundCalving	Binary	Unseasonal calving management	
	VectorFlies	Binary	Flies observed in Pens while sampling	
	VectorBirdDroppings	Binary	Bird droppings observed on surfaces in Pens while sampling	
	VectorBirdNests	Binary	Bird nests observed in Pens while sampling	
	VectorsRodenticide	Binary	Farmer asked if they use rodenticide to control rodents	
	BobbysPresent	Binary	Young veal calves in Pen while sampling	
	ReplacementsPresent	Binary	Young replacement calves in Pen while sampling	

	BeefPresent	Binary	Young beef calves in Pen while sampling	
	BobbysIsolated	Binary	Young veal calves not mixed with other classes (replacement/beef)	
	ReplacementsIsolated	Binary	Replacement calves isolated from other calves	
	BeefIsolated	Binary	Beef calves isolate from other calves	
	BobbysMixedReplacements	Binary	Young veal calves and replacements mixed together	
	BobbysMixedBeef	Binary	Young veal calves and beef calves mixed together	
	ReplacementsMixedBobbys	Binary	Replacement calves and young veal calves mixed together	
	ReplacementsMixedBeef	Binary	Replacement calves and beef calves mixed together	
	BeefMixedReplacements	Binary	Beef calves and replacement calved mixed together	
	BeefMixedBobbys	Binary	Beef calves and young veal calves mixed together	
	BobbyDaysExport	Continuous	Age of young veal calves when exported for veal meat processing	
	ReplacementsDaysPastured	Continuous	Days replacement calves kept in Pens before putting out to pasture	
	BeefDaysPastured	Continuous	Days beef calves kept in Pens before putting out to pasture	
	BeefDaysExport	Continuous	Age of beef calves when exported	

	AdultsWeanlingsMixedCalves	Binary	Do adult cattle or weaned calves have any contact with calves	
	DamBirthing	Categorical	Location where Dams give birth to calves	
	DaysCalvesWithDam	Continuous	Days before calves removed from dams	
	Colostrum	Binary	Directly from dam or mixed colostrum	
	ColostrumMethod	Categorical	Orogastric tube or suckled from dam	
	MilkFresh	Binary	Fresh milk given to calves	
	MilkPowder	Binary	Powdered milk given to calves	
	MilkWaste	Binary	Antibiotic milk given to calves	
	MilkColostrum	Binary	Colostrum fed to calves	
	MilkContainer	Categorical	Teat bucket or Open bucket for feeding	
	MilkFrequency	Categorical	How many times calves fed a day	
	AdlibWater	Binary	Water available for all calves in Pens	
	ConcentrateMeal	Binary	Feed concentrates provided to calves	
	ConcentrateHayStraw	Binary	Hay or straw provided to calves	
	ConcentrateClay	Binary	Clay additive provided to calves to prevent scours	
	ConcentrateOther	Categorical	Variety of other supplements used by farmers for calves	
	ConcentrateStartFeedDay	Continuous	Day farmer started giving calves concentrates	
	ConcentratesBobby	Binary	Young veal calves received concentrate feed	

	WaterSource	Categorical	Source of water on farm	
	EffluentMethod	Categorical	Spread on farmer's pasture or shipped out	
	EffluentFrequency	Continuous	How often effluent spread on pastures	
	Grazier	Binary	Farmer ships out replacement calves to grazier, and then brings back once they grow to heifers	
	FeedPadHerdHome	Binary	Use of feed pad or herd home for adult cattle	

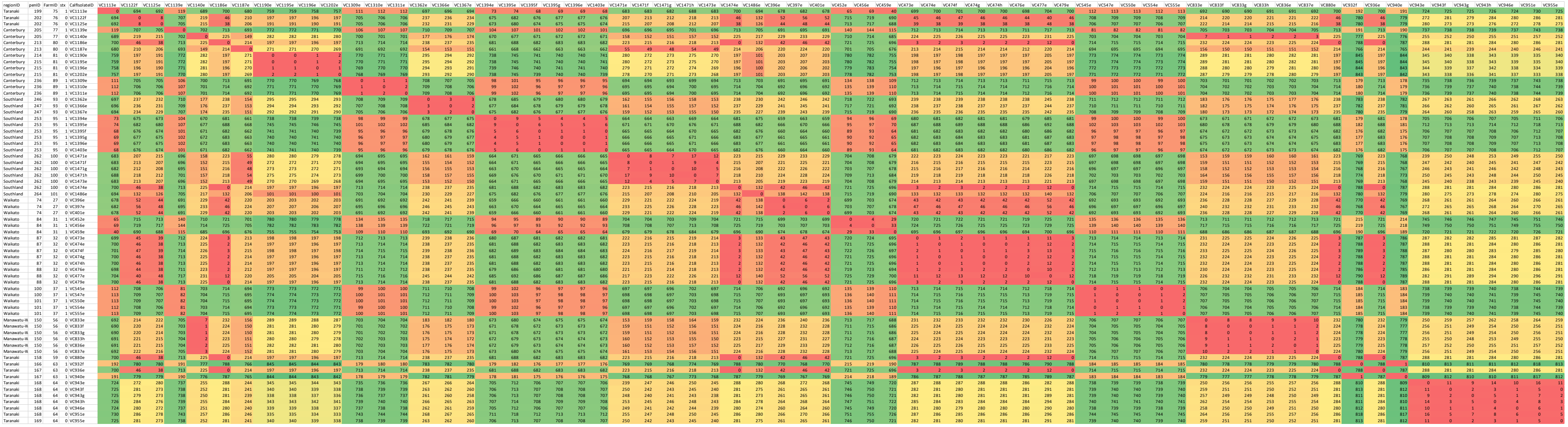

Figure S1: Heatmap of SNP distances between *E. coli* serogroup O26 isolates (n=66); GREEN indicates larger SNP distances (dissimilar isolates), while RED indicates smaller SNP distances (very similar isolates)
